# RETRACTION: SPC25 May Promote Proliferation and Metastasis of Hepatocellular Carcinoma via p53

**DOI:** 10.1002/2211-5463.70162

**Published:** 2025-11-19

## Abstract

**RETRACTION**: F. Chen, K. Zhang, Y. Huang, F. Luo, K. Hu, and Q. Cai, “SPC25 May Promote Proliferation and Metastasis of Hepatocellular Carcinoma via p53,” *FEBS Open Bio* 10, no. 7 (2020): 1261‐1275. https://doi.org/10.1002/2211‐5463.12872.

The above article, published online on 29 April 2020 in Wiley Online Library (wileyonlinelibrary.com), has been retracted by agreement between the journal Editor‐in‐Chief, Miguel De la Rosa; the Federation of European Biochemical Societies; and John Wiley & Sons Ltd., UK. A third party reported that images in Figures 11A, 11B, and 11C had been published in other articles both prior to and after the publication of this article and are also listed as representing different experiments [Ling et al. 2019 (https://doi.org/10.3892/ijo.2019.4926)]; [Ke et al. 2020 (https://doi.org/10.1186/s12935‐020‐01474‐7)]; and [Ling et al. 2021 (https://doi.org/10.18632/aging.203318)]. Additionally, the Migration and Invasion images for si‐SPC25 contain an overlapping section. An investigation by the journal confirmed these instances of image duplication and also discovered that the HepG2 and SMMC‐7721 cell lines discussed in this article are known to be misclassified and contaminated, respectively. The authors did not respond to an inquiry and request for original data by the publisher.

The retraction has been agreed to because the evidence of image duplication across multiple publications, in which the images are reported as different samples by different author groups, fundamentally compromises the editors’ confidence in the results presented. As a result, the Editor‐in‐Chief, FEBS Press, and John Wiley and Sons Ltd. have determined that a retraction is necessary. The authors did not respond to our notice regarding the retraction.

**RETRACTION**: F. Chen, K. Zhang, Y. Huang, F. Luo, K. Hu, and Q. Cai, “SPC25 May Promote Proliferation and Metastasis of Hepatocellular Carcinoma via p53,” *FEBS Open Bio* 10, no. 7 (2020): 1261‐1275. https://doi.org/10.1002/2211‐5463.12872.

The above article, published online on 29 April 2020 in Wiley Online Library (wileyonlinelibrary.com), has been retracted by agreement between the journal Editor‐in‐Chief, Miguel De la Rosa; the Federation of European Biochemical Societies; and John Wiley & Sons Ltd., UK. A third party reported that images in Figures 11A, 11B, and 11C had been published in other articles both prior to and after the publication of this article and are also listed as representing different experiments [Ling et al. 2019 (https://doi.org/10.3892/ijo.2019.4926)]; [Ke et al. 2020 (https://doi.org/10.1186/s12935‐020‐01474‐7)]; and [Ling et al. 2021 (https://doi.org/10.18632/aging.203318)]. Additionally, the Migration and Invasion images for si‐SPC25 contain an overlapping section. An investigation by the journal confirmed these instances of image duplication and also discovered that the HepG2 and SMMC‐7721 cell lines discussed in this article are known to be misclassified and contaminated, respectively. The authors did not respond to an inquiry and request for original data by the publisher.

The retraction has been agreed to because the evidence of image duplication across multiple publications, in which the images are reported as different samples by different author groups, fundamentally compromises the editors’ confidence in the results presented. As a result, the Editor‐in‐Chief, FEBS Press, and John Wiley and Sons Ltd. have determined that a retraction is necessary. The authors did not respond to our notice regarding the retraction.

